# Isoproterenol Induces Vascular Oxidative Stress and Endothelial Dysfunction via a Giα-Coupled β_2_-Adrenoceptor Signaling Pathway

**DOI:** 10.1371/journal.pone.0091877

**Published:** 2014-03-12

**Authors:** Ana P. Davel, Patricia C. Brum, Luciana V. Rossoni

**Affiliations:** 1 Department of Structural and Functional Biology, Institute of Biology, State University of Campinas-UNICAMP, Campinas, SP, Brazil; 2 Department of Physiology and Biophysics, Institute of Biomedical Sciences, University of São Paulo, São Paulo, SP, Brazil; 3 School of Physical Education and Sport, University of São Paulo, São Paulo, SP, Brazil; University of Southampton, United Kingdom

## Abstract

**Objective:**

Sustained β-adrenergic stimulation is a hallmark of sympathetic hyperactivity in cardiovascular diseases. It is associated with oxidative stress and altered vasoconstrictor tone. This study investigated the β-adrenoceptor subtype and the signaling pathways implicated in the vascular effects of β-adrenoceptor overactivation.

**Methods and Results:**

Mice lacking the β_1_- or β_2_-adrenoceptor subtype (β_1_KO, β_2_KO) and wild-type (WT) were treated with isoproterenol (ISO, 15 μg.g^−1^.day^−1^, 7 days). ISO significantly enhanced the maximal vasoconstrictor response (Emax) of the aorta to phenylephrine in WT (+34%) and β_1_KO mice (+35%) but not in β_2_KO mice. The nitric oxide synthase (NOS) inhibitor L-NAME abolished the differences in phenylephrine response between the groups, suggesting that ISO impaired basal NO availability in the aorta of WT and β_1_KO mice. Superoxide dismutase (SOD), pertussis toxin (PTx) or PD 98,059 (p-ERK 1/2 inhibitor) incubation reversed the hypercontractility of aortic rings from ISO-treated WT mice; aortic contraction of ISO-treated β_2_KO mice was not altered. Immunoblotting revealed increased aortic expression of Giα-3 protein (+50%) and phosphorylated ERK1/2 (+90%) and decreased eNOS dimer/monomer ratio in ISO-treated WT mice. ISO enhanced the fluorescence response to dihydroethidium (+100%) in aortas from WT mice, indicating oxidative stress that was normalized by SOD, PTx and L-NAME. The ISO effects were abolished in β_2_KO mice.

**Conclusions:**

The β_2_-adrenoceptor/Giα signaling pathway is implicated in the enhanced vasoconstrictor response and eNOS uncoupling-mediated oxidative stress due to ISO treatment. Thus, long-term β_2_-AR activation might results in endothelial dysfunction.

## Introduction

Activation of the sympathetic system is a common feature in cardiovascular diseases [1]. Acute β-adrenergic activation exerts essential physiological control of cardiovascular systems, increasing cardiac output and inducing vasodilatation. However, overactivation of β-adrenenoceptor (β-AR) induces cardiomyopathy; accordingly, β-AR blockade improves left ventricular function and survival in heart failure patients [2].

The signaling mechanisms associated with β-AR overactivation have been studied in using isoproterenol (ISO)-treated animals [3]. It was demonstrated that ISO treatment induces myocardial oxidative stress [4] and synthesis of proinflammatory cytokines [5], [6]; these mechanisms were also involved in long-term β-AR stimulation-induced cardiac damage, such as cardiac hypertrophy, necrosis and fibrosis. Despite increasing evidence demonstrating the effects of ISO treatment on the heart, little is known about its effects on the vasculature. We previously demonstrated that ISO treatment increased superoxide anion production in the rat aorta, increasing the vasoconstrictor response to the α_1_-adrenoceptor agonist phenylephrine [7], [8]. Oxidative stress associated with altered vascular reactivity was also found in the cerebral arteries of ISO-treated rats, where it mediated cerebrovascular damage [9]. However, the signaling pathway associated with vascular oxidative stress induced by β-AR overactivation has not been elucidated.

Cardiac hypertrophy was shown to be induced by ISO via β_1_-AR signaling [10], [11]. Accordingly, it was demonstrated that ISO induced oxidative stress via β_1_-AR by reducing CuZn-SOD expression in rat myocardium [12]. However, the role of β_2_-AR in the pathophysiology of this model remains unclear. ISO infusion in β_2_-AR knockout mice enhanced the mortality rate and induced more apoptosis in the heart, suggesting a protective role of β_2_-AR [11]. In contrast, prolonged use of β_2_-AR agonists was detrimental in both animals and humans [13], [14]. According, mice overexpressing β_2_-AR showed cardiac inflammation and failure, associated with NADPH oxidase-induced oxidative stress [15]. In blood vessels, early stimulation of β_1_-, β_2_- and β_3_-AR, in lesser or bigger extension, can induce vasodilatation [16], [17]. Although, ISO-induced β-AR overactivation leads to oxidative stress and high vascular contractility [8]. It was shown that the β_2_-AR might signal by both Gs and Gi α-subunit protein stimulating different signaling cellular pathways [18]. However, the individual role of β-AR subtypes underlying the vascular effects of β-AR overactivation has not been investigated. Therefore, the aim of this study was to investigate the β-AR subtype(s) involved in the vascular effects induced by ISO treatment, as well as the mechanisms underlying these alterations.

## Materials and Methods

This investigation was approved by the Ethical Committee for Animal Research of the Institute of Biomedical Sciences of the University of Sao Paulo (permit number: 82/2) and it conforms with the guidelines for ethical conduct in the care and use of animals established by the Brazilian Society of Laboratory Animal Science (SBCAL/COBEA).

### Mice

Male mice (4 month-old) lacking functional β_1_- or β_2_-ARs and congenic C57BL/6J or FVB/N background strains were used in this study [19], [20]. Animals were maintained on a 12/12 h light/dark cycle in a temperature-controlled environment (23°C) with free access to standard laboratory chow and tap water. Knockout (KO) and wild-type (WT) mice were randomly treated daily with ISO (15 μg.g^−1^.day^−1^, sc, suspended in 50 μL soy bean oil) or vehicle for 7 days. At the end of the treatment, animals were killed by decapitation and heart and aorta were carefully removed and processed according to the desired experiments. The ratio of the left ventricle weight to tibia length was used as an index of ventricular hypertrophy and confirmed the efficacy of ISO treatment in WT mice.

### Vascular reactivity study

Cylindrical segments (rings) of the thoracic aorta (2 mm in length), free of connective tissue, were mounted in an isolated tissue chamber containing Krebs-Henseleit solution (in mM: 118 NaCl, 4.7 KCl, 25 NaHCO_3_, 2.5 CaCl_2_-2H_2_O, 1.2 KH_2_PO_4_, 1.2 MgSO_4_-7H_2_O, 11 glucose, and 0.01 EDTA) gassed with 95% O_2_ and 5% CO_2_. Rings were maintained at a resting tension of 0.5 g at 37°C at pH 7.4 as previously described [21]. Isometric tension was recorded using an isometric force transducer (Letica TRI 210, Spain) connected to an acquisition system (MP100, BiopacSystems, USA).

After a 60 min equilibration period, aortic rings were exposed to 125 mM KCl to assess the maximal tension. Endothelial integrity was tested by acetylcholine-induced relaxation (10 μM, Sigma-Aldrich, Germany) in aortic rings that were contracted with phenylephrine (∼0.1 μM, Sigma-Aldrich). A relaxation response to acetylcholine larger than 50% was considered to demonstrate functional integrity of the endothelium. After a washout period, concentration-response curves to the α_1_-adrenoceptor agonist phenylephrine (0.1 nM–10 μM) were obtained.

To evaluate the role of NO and superoxide anion in the vasoconstrictor response to phenylephrine, some aortic rings were pre-incubated for 30 minutes with the nonselective nitric oxide synthase (NOS) inhibitor N-nitro-L-arginine methyl ester (L-NAME, 100 μM, Sigma-Aldrich) or with superoxide dismutase (SOD, 150 U/mL, bovine erythrocyte, Sigma-Aldrich). In addition, some aortic rings from WT and β_2_KO were incubated for 1 h with pertussis toxin to inactivate Giα protein (PTx, 0.5 μg/mL, Sigma-Aldrich) [22], [23] or for 30 min with MEK (MAPKK) inhibitor PD 98,059 (1 μM, Sigma-Aldrich), to inhibit phosphorylation of ERK1/2 [24] before the concentration-response curves to phenylephrine were assessed. Time controls for each drug pre-incubation were performed. Vasoconstrictor responses to phenylephrine were expressed as a percentage of the contraction produced by 125 mM KCl.

Our results demonstrated that the changes induced by ISO treatment on ventricular morphometry and vascular function did not differ between C57BL/6J and FVB/N strains (data not shown). Thus, in the present study we used the inbred FVB/N strain as the wild-type mice.

### Western blot analysis

Total protein extract was obtained from isolated aortas homogenized in cold RIPA lysis buffer (Amersham, N.J., USA) containing PMSF (1 mM) and Na_3_VO_4_ (1 mM). The homogenates were centrifuged (1,500 g for 20 min at 4°C) and the supernatants were isolated. The microsomal fraction of aortic tissue was obtained from a pool of three aortas homogenized in ice-cold sucrose-Tris-EDTA buffer (Tris 50 mM, sucrose 250 mM, and EDTA 1.0 mM, pH = 7.4). The initial centrifugation was 10,000 g for 10 min at 4°C, and then the supernatant was centrifuged at 100,000 g for 60 min. The pellet representing the microsomal fraction was resuspended in Tris-EDTA. To investigate eNOS dimer:monomer ratio, aortas were lysed in buffer (50 mmol/L Tris-HCl pH = 8.0; 0.2% Nonidet P-40; 180 mmol/L NaCl; 0.5 mmol/L EDTA; 25 mmol/L phenylmethylsulphonyl fluoride; 0.1 mmol/L dithiothreitol; and protease inhibitors). The protein extracts were quantified in each sample using a BCA Protein Assay Kit (Thermo Fisher Scientific Inc., Mass., USA).

Total and microsomal protein extract (40 μg and 15 μg, respectively) were electrophoretically separated by 10% SDS-PAGE. To analyse eNOS dimerization, non-boiled samples (40 μg) were resolved by 6% SDS-PAGE at 4°C [25]. Then, proteins were transferred to polyvinylidene difluoride membranes (Amersham, USA) overnight at 4°C. The transfer used a Mini Trans-Blot Cell system (Bio-Rad, USA) containing 25 mmol/L Tris, 190 mmol/L glycine, 20% methanol, and 0.05% SDS as previously described [21]. After blockade of nonspecific sites with 5% nonfat dry milk, membranes containing total protein extract were incubated overnight at 4°C with the following primary antibodies: anti-Giα-1,2 protein (1∶2,000; Upstate, USA), anti-Giα-3 protein (1∶2,000; Upstate), anti-ERK 1/2 (1∶1,000, Cell Signaling), anti-phospho (Thr202/Tyr204)-ERK1/2 (Cell Signaling; 1∶1,000), anti-p38 MAPK (1∶1,000, Cell Signaling), anti-phospho (Thr180/Tyr182)-p38 MAPK (Cell Signaling; 1∶1,000), anti-α-actin antibody (1∶3,000, Sigma-Aldrich). Membranes containing non-boiled samples were incubated with anti-eNOS (1∶1,000, BD Transduction Laboratories, USA). The protein content of α-actin was used as an internal control. Membranes containing proteins from the microsomal fraction were incubated overnight with primary antibodies against β_1_-, β_2_- and β_3_-adrenoceptors (1∶500; Santa Cruz Biotechnology). Reversible Ponceau staining (1%, Amresco) was used to check equal loading of microsomal fraction gels.

After washing (10 mM Tris, 100 mM NaCl, and 0.1% Tween 20), membranes were incubated for 2 hours with a peroxidase-conjugated IgG antibody according to each primary antibody used. Immunocomplexes were detected using an enhanced horseradish peroxidase-luminol chemiluminescence system (ECL Prime, GE Healthcare) and subjected to autoradiography (Hyperfilm ECL, Amersham). Signals on the immunoblot were quantified with ImageJ software (NIH, USA).

### Reactive oxygen species (ROS) generation

The oxidative fluorescent dye hydroethidine was used to evaluate the *in situ* production of ROS [8], [21]. Briefly, transverse aortic sections (10 μm) obtained in a cryostat were incubated at 37°C for 10 min with phosphate buffer. Fresh buffer containing hydroethidine (5 μM) was topically applied to each tissue section and the slides were incubated in a light-protected, humidified chamber at 37°C for 30 min. Some aortic slices were incubated with phosphate buffer containing apocynin (30 μM, 30 min), PEG-SOD (150 U/mL, 30 min), L-NAME (100 μM, 30 min), PTx (0.5 μg/mL, 1 hour) or vehicle (deionized water; time controls). Negative control sections received the same volume of phosphate buffer without hydroethidine. Images were obtained with an optical microscope (Eclipse 80i, Nikon, Japan) equipped with a rhodamine filter and camera (DS-U3, Nikon, Japan) using a 20× objective.

### Statistical analysis

Results were expressed as the mean ± SEM, and *N* represented the number of mice used in each set of experiments. Differences in the area under the concentration-response curves (AUC) in the absence (control) and presence of PTx or PD 98,059 were calculated using GraphPad Prism program. The differences were expressed as a percentage of the AUC of the corresponding control treatment.

Data were analyzed by a 2-way ANOVA followed by Bonferroni post-hoc correction or Student's *t*-test using the GraphPad Prism. Differences between groups were considered significant at *P*<0.05.

## Results

ISO treatment for 7 days induced a significant increase in the weight of the left ventricle of wild-type (WT) mice (∼24%) and β_2_-AR knockout mice (∼28%). No increase was observed in β_1_-AR knockout mice. This result was in line with previous studies and confirmed the ability of ISO to induce cardiac hypertrophy via β_1_-AR activation [11].

### β_2_-AR mediates the increased vascular reactivity to phenylephrine induced by ISO treatment

**Figure 1:**
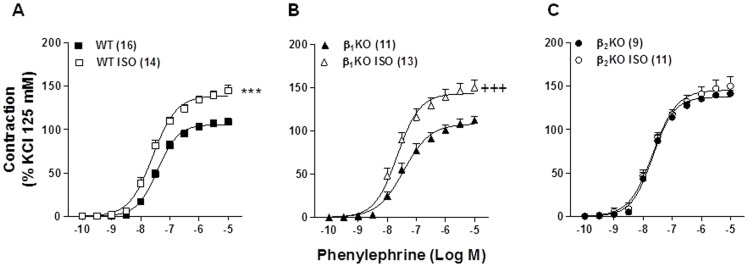
Knockout of β_2_-AR prevent increase of phenylephrine contractile response induced by isoproterenol in aorta. 7-day isoproterenol treatment (ISO) increased the vasoconstrictor response to phenylephrine in isolated thoracic aorta from wild-type (WT) (A) and β_1_-KO (B) mice. This effect was abolished in β_2_-KO mice (C). The contraction response is expressed as a % of the contraction to KCl (125 mM). The number of animals used in each group is indicated in parenthesis. Values are presented as the mean ± SEM. Significance was assessed with a 2-way ANOVA: ***p<0.0001 *vs.* WT; ^+++^p<0.0001 *vs*. β_1_KO.

The contractile response induced by KCl (125 mM) was not modified by ISO treatment in WT (WT: 0.53±0.03 g *vs*. WT/ISO: 0.60±0.04 g, p>0.05; *t*-test), β_1_-AR knockout mice (β_1_KO: 0.52±0.03 g *vs.* β_1_KO/ISO: 0.59±0.04 g, p>0.05; *t*-test) or β_2_-AR knockout mice (β_2_KO: 0.59±0.03 g *vs.* β_2_KO/ISO: 0.68±0.04 g, p>0.05; *t*-test). ISO treatment for 7 days enhanced the phenylephrine-induced vasoconstrictor response in the aortas of WT mice (Figure 1A); the maximal contractile effect (Emax) was increased by 33% (WT: 108.3±4.4% *vs*. WT/ISO: 144.8±6.0% to KCl 125 mM, p<0.0001; *t*-test) without significant changes in potency. Similar results were observed in the aortas of β_1_KO mice (Figure 1B); ISO increased the Emax by 36% (β_1_KO: 111.8±5.0% *vs.* β_1_KO/ISO: 153.2±8.9% to KCl 125 mM, p<0.0001; *t*-test). However, a lack of functional β_2_-ARs prevented the changes caused by ISO in phenylephrine-induced contraction, and no differences in the Emax were observed between untreated and ISO-treated groups (Figure 1C).

### Lack of functional β_2_-AR prevents ISO-induced NO impair and high superoxide anion production in aorta

**Figure 2:**
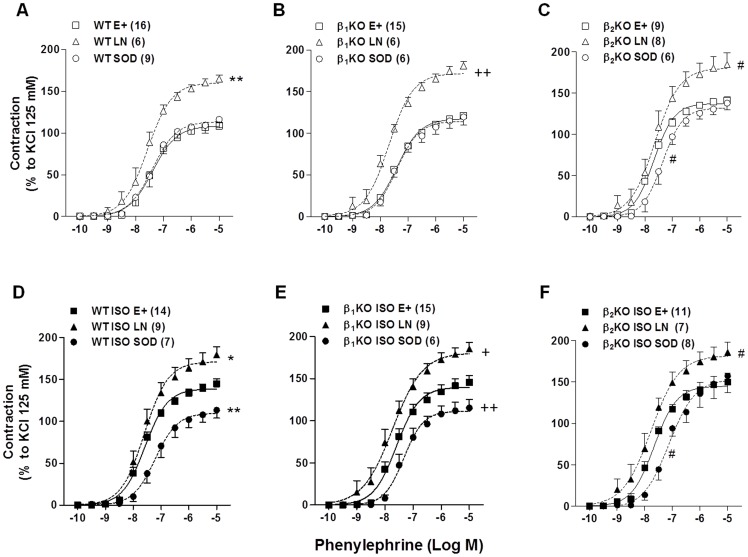
Role of superoxide anion and NOS in the vascular effect of isoproterenol treatment. Effect of L-NAME (LN, 100 μM) or superoxide dismutase (SOD, 150 U/mL) on the concentration-response curves to phenylephrine of vehicle- (open symbols) or 7-day isoproterenol-treated (ISO, close symbols) aortic rings from wild-type (A, D), β_1_KO (B, E) and β_2_KO (C, F) mice. The contraction response is expressed as a % of the contraction to KCl (125 mM). Values are presented at the mean ± SEM. E+ =  intact endothelium. The number of animals used in each group is indicated in parenthesis. Significance was assessed using a 2-way ANOVA: *p<0.05, **p<0.01 *vs*. WT E+; ^+^p<0.05, ^++^p<0.01 *vs.* β_1_KO E+; ^#^p<0.05 *vs*. β_2_KO E+.

Incubation with L-NAME potentiated the phenylephrine-induced contraction in aortas from all groups evaluated (Figure 2). In aortas from WT and β_1_KO mice, L-NAME increased the Emax to phenylephrine by 56 and 63%, respectively (Figure 2A and 2B), and in aortas from ISO-treated WT and β_1_KO mice, L-NAME increased this contractile response by only 24 and 20%, respectively (Figures 2D and 2E). There was a reduction in the magnitude of the L-NAME effect in ISO-treated WT and β_1_KO mice (L-NAME *vs*. basal: p<0.05, 2-way ANOVA; Figures 2D and 2E) in comparison to non-treated WT and β_1_KO groups (L-NAME *vs*. basal: p<0.001, 2-way ANOVA; Figures 2A and 2B). SOD did not modify phenylephrine contraction in untreated WT and β_1_KO mice (Figures 2A and 2B), but significantly reduced this response in aortas from WT and β_1_KO mice treated with ISO (p<0.01, 2-way ANOVA; Figures 2D and 2E), reversing the increase in phenylephrine contraction induced by ISO (Figures 2A and 2B). Thus, the effects of ISO on vascular reactivity to phenylephrine were very similar between WT and β_1_KO aortas, as well the effects of L-NAME and SOD incubation.

Previous data from our group demonstrated that knockout of β_2_-AR affected the phenylephrine response in aortic rings [21]. In line with this, we observed that the increase in phenylephrine response induced by L-NAME in aortas from untreated β_2_KO was significant (L-NAME *vs.* basal: p<0.05, 2-way ANOVA), but in minor magnitude then WT (Figure 2A and 2C); and SOD reduced the contractile response to phenylephrine in this vessel without changes the in Emax (Figure 2C). The treatment of β_2_KO mice with ISO did not change the magnitude of the effects of L-NAME or SOD compared with non-treated β_2_KO mice (Figures 2C and 2F).

Together, these results reinforce our previous studies [7], [8], [26], suggesting that ISO treatment induces changes in vascular reactivity to phenylephrine that are associated with oxidative stress. Furthermore, we add new data that these adjustments seem to be dependent of the presence of functional vascular β_2_-AR.

### Expression of β-AR subtypes is not affected by ISO treatment

**Figure 3:**
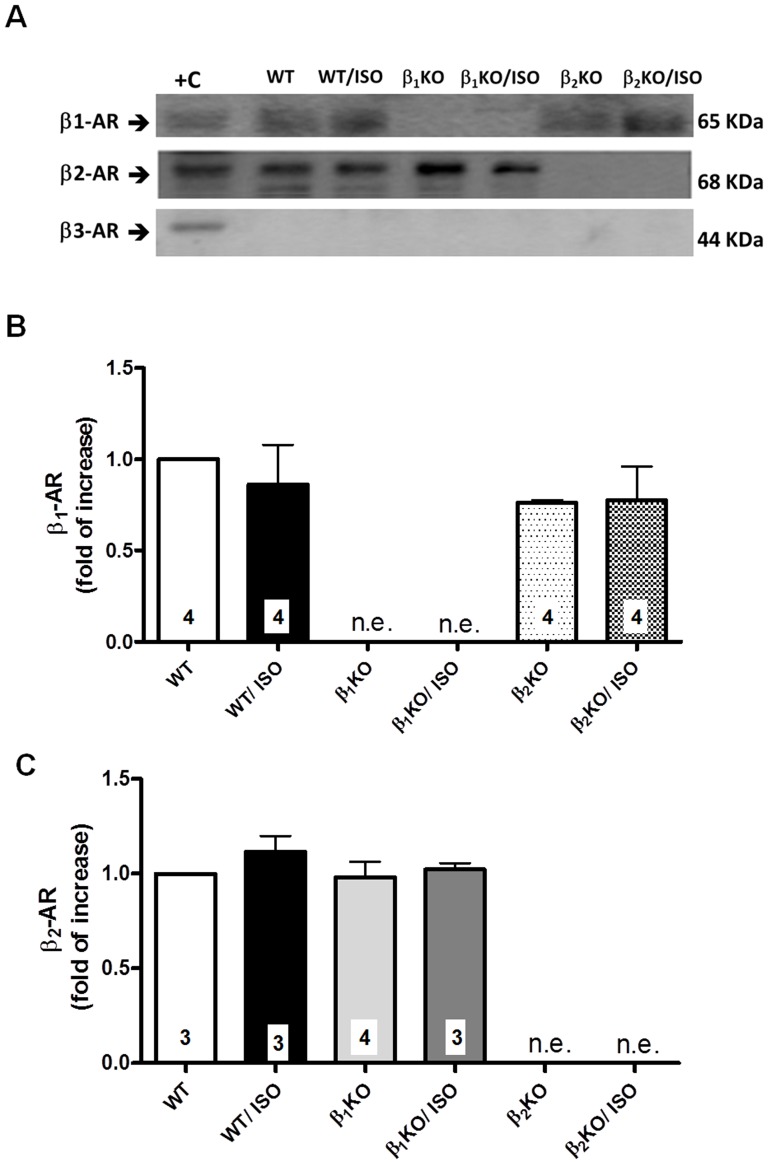
Aortic β-AR subtypes expression. Protein expression of β_1_- β_2_- and β_3_-adrenoceptors (AR) evaluated in the membrane fraction of aortas from WT, β_1_KO and β_2_KO mice treated for 7 days with vehicle or isoproterenol (ISO). (A) Representative Western-blot autoradiographs for each β-AR subtype in membrane preparations of aorta and positive controls (+C: heart for β_1_-AR; skeletal muscle for β_2_-AR; adipose tissue for β_3_-AR). Densitometric quantification was evaluated for β_1_- (B) and β_2_-AR (C) but not for β_3_-AR, as this subtype was not expressed (n.e.) in the mouse aorta. The number of samples analyzed (pool of 3 aortas in each sample) is indicated in the bar for each group. Values (mean ± SEM) are expressed the fold-change in β-AR expression compared to the WT. Significance was assessed using a 2-way ANOVA.

The membrane fraction of aortas from WT animals expressed both β_1_- and β_2_-ARs, while the β_3_-AR subtype was not detected (Figure 3A, 3B and 3C). As expected, no significant staining for the β_2_-AR subtype was observed in aortas of β_2_KO mice, and β_1_-AR was not detected in aortas of β_1_KO mice (Figure 3A, 3B and 3C). ISO treatment did not modify the expression of the β_1_- or β_2_-AR in any group, and the β_3_-AR protein expression remained undetected (Figure 3A, 3B and 3C).

### ISO treatment enhanced Giα-3 protein expression and ERK1/2 phosphorylation, whereas reduced eNOS dimerization in aortas from WT but not β_2_KO mice

**Figure 4:**
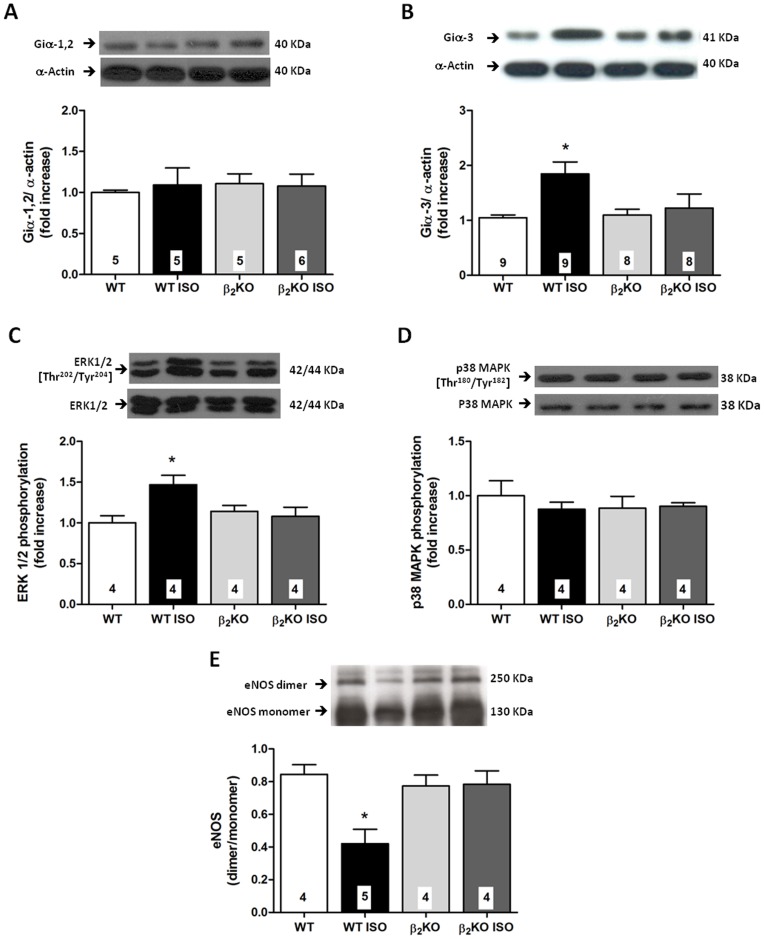
Isoproterenol treatment induces β_2_-AR-Gi-ERK1/2 pathway activation and eNOS uncoupling. Protein expression of Giα-1,2 (A), Giα-3 (B), ERK 1/2 phosphorylated at Thr^202^ and Tyr^204^ (C), p38 MAPK phosphorylated at Thr^180^ and Tyr^182^ (D) and eNOS protein dimerization (E) in aortas from control and 7-day isoproterenol-treated (ISO) wild-type (WT) and β_2_KO mice. The top panels in each graph represent typical Western-blot autoradiographs. Giα protein expression was normalized to the α-actin content in each sample, and phosphorylated ERK 1/2 and p38 MAPK were normalized to the total content of ERK 1/2 and p38 MAPK, respectively, and these results were expressed as the fold-change compared to WT aorta. eNOS dimerization was expressed as ratio of dimer:monomer band intensity. The number of animals used in each group is indicated in the bars. Values are presented as the mean ± SEM. Significance was assessed using a 2-way ANOVA: *p<0.05 *vs.* WT.

ISO treatment significantly enhanced the expression of Giα-3 protein in aortas from WT but not β_2_KO mice (Figure 4B). No changes in Giα-1 or -2 protein levels were found among the groups (Figure 4A). The total protein expression of ERK 1/2 and p38 MAPK was not modified by the lack of β_2_-AR or by ISO treatment. However, ISO enhanced the phosphorylation of ERK 1/2 at residues Thr202/Tyr204 (Figure 4C) but did not alter the phosphorylation of p38 MAPK (Figure 4D). We further investigated whether eNOS protein dimerization was altered by β-AR overactivation. After ISO treatment, the ratio of eNOS dimer to monomer was significantly lower in aortas from WT mice; whereas the ISO effect was prevented in β_2_KO mice (Figure 4E). It was related to a reduction of 62% in the abundance of the dimeric active form of eNOS in WT ISO group compared with non-treated WT, that not occur in β_2_KO mice.

### Implication of β_2_-AR/Giα signaling on vascular oxidative stress and increased response to phenylephrine following ISO treatment

**Figure 5:**
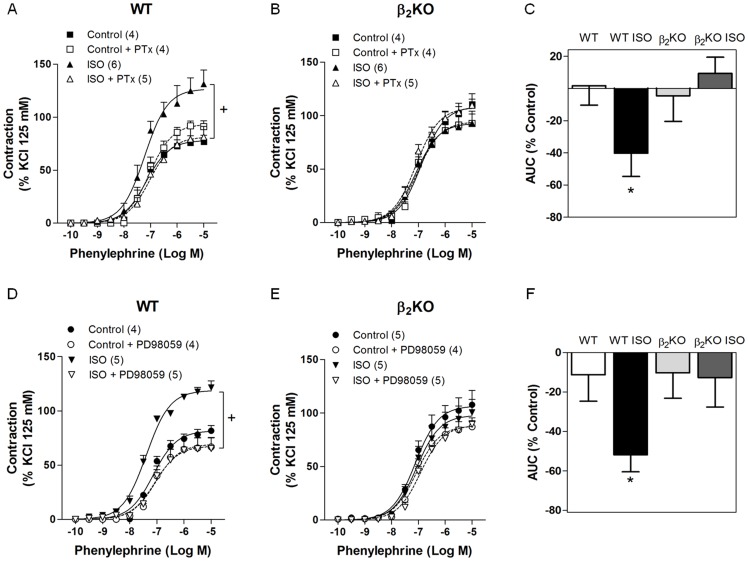
Inhibition of Giα protein or ERK1/2 activation reversed hypercontractility to phenylephrine induced by β-AR overactivation in aorta of wild-type, but not in β_2_KO mice. Effect of pertussis toxin (PTx, 4 μM) and PD98,059 (1 μM) on the concentration-response curves to phenylephrine in aortic rings of wild-type (WT) (A, D) and β_2_KO (B, E) mice treated for 7 days with vehicle or isoproterenol (ISO). The contraction response is expressed as a % of the contraction to KCl (125 mM). Bar graphs show differences in the area under the concentration-response curve (AUC) in the presence or absence of PTx (C) or PD98,059 (F) in WT and β_2_KO mice treated or not with ISO. Values are presented as the mean ± SEM. The number of animals used in each group is indicated in parenthesis. Significance was assessed using a 2-way ANOVA: ^+^p<0.05 *vs.* WT ISO; *p<0.05 *vs.* WT.

Inhibiting Giα protein activity with the pertussis toxin or ERK1/2 phosphorylation with PD 98,059 abolished the increased contraction to phenylephrine in ISO-treated WT mice, reducing the response to control WT levels (Figure 5A and 5D, respectively). Despite these effects on aortas from ISO-treated WT mice, pertussis toxin or PD 98,059 did not change the phenylephrine contraction in non-treated WT (Figure 5A and 5D) or in β_2_KO mice regardless of ISO treatment (Figure 5B and 5E). The difference in AUC of the concentration-response curves to phenylephrine evaluated in the presence and absence of pertussis toxin (Figure 5C) and PD 98,059 (Figure 5F) revealed significant participation of Giα protein and p-ERK1/2 pathway in the phenylephrine response of aorta from ISO-treated WT mice, but not in β_2_KO.

**Figure 6:**
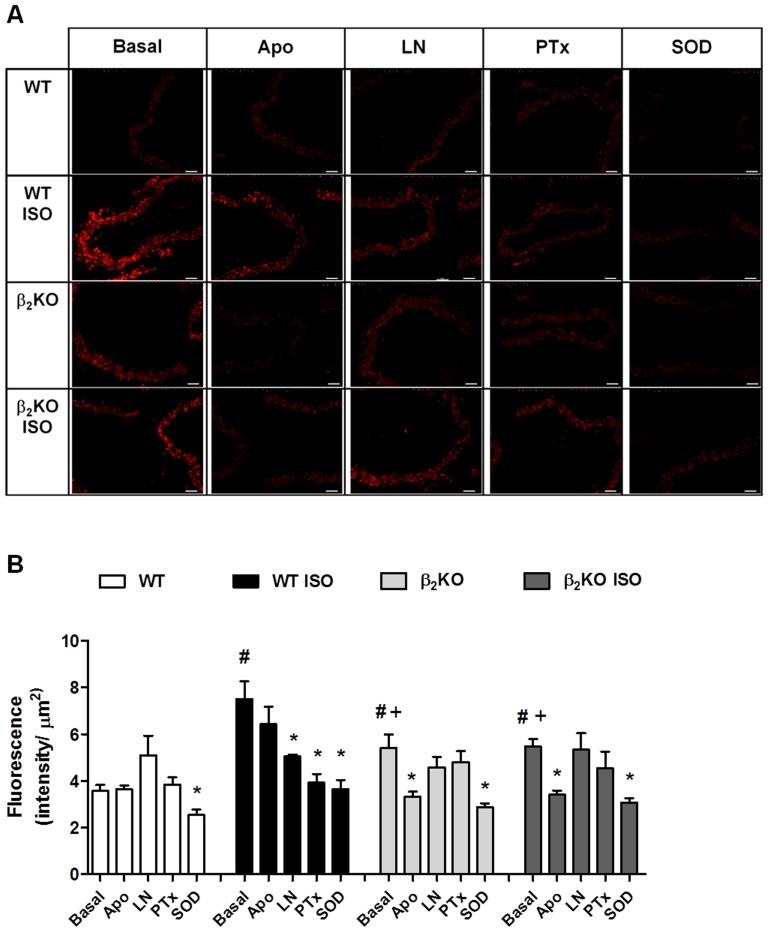
Giα protein activity mediates the vascular oxidative stress induced by isoproterenol. Panel A shows representative fluorographs of microscopic sections of thoracic aorta from wild-type (WT) and β_2_KO mice treated for 7 days with vehicle or isoproterenol (ISO). Vessels were labeled with the oxidative dye hydroethidine, which produces a red fluorescence when oxidized to ethidium bromide. Panel B shows the densitometric analysis of the ethidium-bromide-positive nuclei evaluated under basal conditions or incubated with apocynin (APO, 30 μM), L-NAME (LN, 100 μM), PTx (4 μM) or superoxide dismutase (SOD, 150 U/mL). The fluorescence signal was evaluated as the intensity of fluorescence per pixel normalized by vessel area. Values are presented as the mean ± SEM. N = 4–7 animals in each group. Significance was assessed using a 2-way ANOVA: *p<0.05 *vs*. respective basal values for each group; ^#^p<0.05 *vs*. basal WT value; ^+^p<0.05 *vs*. ISO-treated WT value.

Reactive oxygen species production was evaluated *in situ* by quantification of hydroethidine fluorescence emission. Basal oxidative stress was observed in aortic slices from ISO-treated WT mice compared to control WT mice (Figure 6A and 6B). A minor but significant enhancement of hydroethidine fluorescence was observed in β_2_KO mice, and this fluorescence was not modified by ISO treatment. Incubation with L-NAME or pertussis toxin significantly reduced the fluorescence to hydroethidine only in ISO-treated WT aortas, normalizing the oxidative stress in this group (Figure 6A and 6B). In contrast, incubation with apocynin reduced the reactive oxygen species only in β_2_KO mice; this was true for both ISO-treated and untreated β_2_KO mice (Figure 6A and 6B). SOD incubation significantly reduced the fluorescence to hydroethidine, abolishing the differences between all groups studied (Figure 6A and 6B).

## Discussion

Sustained sympathetic activation leads to myocardial hypertrophy, which is considered a hallmark of β-AR overstimulation. This precedes heart failure, highlighting the clinical relevance of the sympathetic system. In addition, sustained sympathetic activation in cardiovascular disease is characterized by elevated baseline vascular tone and impaired NO bioavailability [27], [28]. However, the mechanism that leads to these alterations in the vasculature remains unclear. In the present study, we show that the β_2_-AR subtype mediates ISO-induced changes in vascular tone via the coupled Giα pathway. This effect was associated with sustained β_2_-AR activation uncoupling NOS that enhances superoxide anion generation and thereby decreases NO bioavailability.

It is known that acute activation of β-ARs can directly induce vasodilatation in smooth muscle cells via a Gs protein/adenylyl cyclase/cAMP pathway. However, little is known about the mechanisms associated with the effects of long-term β-AR activation in vascular cells. Previous studies suggested that ISO-induced β-AR overactivation leads to alterations in vascular tone depending on the vessel type [8], [9], [26], [29], [30]. In murine aortas, ISO treatment enhances the contractile response to the α_1_-adrenoceptor agonist phenylephrine and is associated with elevated ROS generation and impaired NO bioavailability [8], [30]. In this study, we determined the β-AR subtype as well as the downstream mechanisms involved in this effect.

We observed that ISO treatment enhanced the phenylephrine-induced contraction in aortas from both WT and β_1_KO mice. However, this effect was not observed in β_2_KO mice. These data suggest that the hypercontractile aortic phenotype induced by sustained β-AR activation is mediated via the β_2_-AR. Noteworthy, the aorta from β_2_KO mice presented increased contraction to phenylephrine before ISO treatment. Although the contractility is increased in these vessels, L-NAME incubation significantly increased the phenylephrine-induced contraction ISO- and vehicle-treated β_2_KO mice; it suggested that even the contractility of β_2_KO mice aorta is higher than in WT, it not reached maximal contractility capacity and can be responsible to a stimuli and/or injury that could alter vascular contractility. Previously we demonstrated that β_2_-AR deficiency enhances aortic contractility to phenylephrine associated to NADPH oxidase-derived superoxide anion generation [21]. This evidenced the crucial physiological role of β_2_-AR in the maintenance of vascular tone and redox status. On the other hand, the current study revealed that overstimulation of vascular β_2_-AR has a pathological effect and could serve as a mechanism of vascular injury via Gαi/MAPK-dependent signaling pathway. In line with this possible pathological role of β_2_-AR when overstimulated in vasculature, a previous study by Xu Q *et al*. [15] observed that transgenic mice overexpressing β_2_-AR showed enhanced superoxide anion production, which activated p38 MAPK and contributed to cardiac remodeling and failure.

Consistent with the role of overactivated β_2_-AR in cardiac oxidative stress, we also observed that the ISO-induced ROS generation was virtually abolished in aortas from β_2_KO mice, indicating that the overactivation of β_2_-ARs induced vascular oxidative stress. The vascular oxidative stress induced by ISO seemed to be related to enhanced superoxide anion levels; incubation with superoxide dismutase normalized both the contractile response to phenylephrine and the high fluorescence to hydroethidine in aortas from ISO-treated WT mice. In addition, a previous study from our group demonstrated that SOD content in aortas from ISO-treated rats was enhanced, which counteracted the elevated superoxide generation induced by β-AR overactivation [8].

Enhanced production of superoxide anion is involved in the pathogenesis and complication of many cardiovascular diseases by reducing NO bioavailability. In addition, peroxynitrite and hydroxyl radicals are produced; these mechanisms induce endothelial dysfunction [31], [32]. The basal release of NO involved in the control of vascular tone can be estimated by quantifying the increase in vascular tone by NOS inhibitors like L-NAME [33]. Therefore, we evaluated the effect of L-NAME incubation on the vasoconstrictor response to phenylephrine in aortas from WT, β_1_KO and β_2_KO mice treated or untreated with ISO. We observed that L-NAME incubation showed minor contractile effect on aortas from ISO-treated WT and β_1_KO mice compared to their respective non-treated control groups. These results suggested that control of vascular tone was impaired by a loss of aortic NO bioavailability. However, this effect of L-NAME was not observed in ISO-treated β_2_KO mice compared with non-treated mice. In addition, after L-NAME incubation, there were no significant differences in the phenylephrine-induced contraction among aortas from all groups evaluated. Together, these data suggest that the β_2_-AR subtype mediated the impairment of NO release and the altered vascular tone that were induced by chronic ISO treatment; while the β_1_-AR subtype was not involved. This effect was not expected as studies have demonstrated that acute β_2_-AR activation stimulated NO synthesis in endothelial cells of various vessel types via eNOS activation [34]–[36]. However, overstimulated β_2_-AR seemed to induce a signaling pathway distinct from the one involved in acute vasodilatation. Together, the present data suggest that overactivation of β_2_-AR evoked adverse effects on vascular tissue.

The β_2_-AR couples to both Gs and Gi protein α-subunits, stimulating distinct signaling pathways [18], [37]. Acute activation of β_2_-AR in mouse pulmonary endothelial cells induces eNOS-derived NO production via the Gi-Scr kinase-dependent pathway [38]. However, it is not known if the β_2_-AR/Gi pathway is involved in the vascular effects of sustained β-AR activation. Three distinct proteins, Giα-1, -2 and -3, have been cloned [39], and all three isoforms have been implicated in adenylyl cyclase inhibition [40]. We observed for the first time that ISO treatment significantly elevated the expression of Giα-3 in the vasculature of WT mice in a manner dependent on the presence of functional vascular β_2_-AR.

The involvement of Giα protein signaling in vascular function has been studied using pertussis toxin it *in vivo* and *in vitro*. Pertussis toxin ADP-ribosylates the Gi protein α-subunit, which contains a cystein residue near the carboxy terminus and thus inactivates its activity [41], [42]. Here, both the enhanced response to phenylephrine and the oxidative stress found in aortas from ISO-treated WT mice were pertussis toxin-sensitive. In contrast, Gi protein inhibition did not affect aortas from untreated WT mice or ISO-treated or untreated β_2_KO mice. These results indicate that the Giα pathway activated by ISO in the vasculature was dependent on β_2_-AR. Previous studies found enhanced levels of Giα-2 and -3 protein and mRNA in aortas from hypertensive animals [43], [44]. Treatment with pertussis toxin reduced the contractile response to norepinephrine on conduct arteries of spontaneously hypertensive rats (SHR). In addition, superoxide anion production was decreased in the vascular smooth muscle cells of the SHR rats treated with pertussis toxin [42], [45]. Together, these data suggest a potential beneficial effect of selective inhibition of the β_2_-AR/Giα signaling pathway in the vasculature. In particular, β_2_-AR/Giα inhibition could be of benefit in cardiovascular diseases characterized by high sympathetic tone, such as essential hypertension and heart failure.

Superoxide anion generation was inhibited by L-NAME only in aortas from ISO-treated WT, without effect in control WT as well as in β_2_KO mice with or without ISO treatment. In line with this finding, we also observed a relative reduction in the abundance of the active dimeric form of eNOS in ISO-treated WT. By contrast, dimerization of eNOS in aortas from β_2_KO treated or not with ISO was similar to observed in control WT. It is known that reduced eNOS dimerization is associated with impaired NO bioavailability and increased superoxide anion production [46], and previous reports demonstrated that the superoxide anion generation by eNOS is inhibitable by L-NAME incubation [47], [48]. Taken together, these results suggest that enhanced superoxide anion production induced by ISO is derived from uncoupled eNOS and is dependent on β_2_-AR. In line with the role of eNOS uncoupling mediating the vascular effect of β-AR overactivation, L-arginine incubation reduced the increased contractile response to phenylephrine in aortic rings from ISO-treated rats [8]. Similar to the effect observed for L-NAME, pertussis toxin decreased hydroethidine fluorescence only in aortas from ISO-treated WT mice, suggesting eNOS uncoupling induced by β-AR overactivation is associated with Giα protein signaling. Furthermore, there was no effect of apocynin on the ISO-induced vascular oxidative stress in aortic mice, which reinforce that vascular NADPH oxidase was not involved in this effect of ISO. Of note, lack of β_2_-AR induced a mild oxidative stress in the aorta that was reversed by apocynin. This result agreed with a previous study that reported an antioxidant role of constitutive β_2_-ARs, in which inhibition of NADPH oxidase contributed to the maintenance of vascular tone [21]. Conversely, L-NAME or pertussis toxin did not affect the DHE fluorescence in vessels from β_2_KO mice, treated or untreated with ISO. Taken together, these observations suggested that the β_2_-AR/Giα pathway leads to eNOS uncoupling during ISO-induced β-AR overactivation. This role of β_2_-AR is in addition to its physiological role inhibiting NAPDH oxidase. The importance of this mechanism is raised by the fact that therapeutic interventions that could improve eNOS uncoupling were proposed to ameliorate endothelial dysfunction in many cardiovascular diseases associated with β-adrenergic overstimulation [53].

Gi protein-coupled β_2_-AR induces the activation of MAPKs [18]. It was previously demonstrated that long-term ISO stimulation of β-ARs enhanced phosphorylated ERK 1/2 in the heart and cerebral arteries. This was concomitant with a time-dependent reduction in PKA activity [49]. We demonstrated here that 7-day ISO treatment enhanced phosphorylated (p)-ERK 1/2 in the mouse aorta. This effect was blocked in β_2_KO mice, evidence for the first time that β_2_-AR overstimulation activates ERK 1/2 in the vasculature. Considering that the activated ERK 1/2 signaling led to eNOS uncoupling [50], the present results strongly suggested that overstimulation of the β_2_-AR/Giα/p-ERK 1/2 signaling pathway mediated the oxidative stress and reduced NO bioavailability induced by ISO treatment. These results provided a new mechanism underlying superoxide anion generation from eNOS uncoupling, which may have an important role in the pathogenesis of endothelial dysfunction in diseases secondary to sympathetic overactivation [51], [52]. Reinforcing this hypothesis, it was found that the inhibition of p-ERK1/2 normalized the hypercontractility to phenylephrine observed in aortas from ISO-treated WT mice; whereas this inhibitor did not show significant effect on the phenylephrine response of aortas from WT, β_2_KO and ISO-treated β_2_KO mice. Thus, these functional results associated with the increased p-ERK1/2 protein expression suggest that an up-regulated p-ERK1/2 pathway is involved in the ISO effect to enhance vascular contractility to phenylephrine associated with reduced NO bioavailability, depending on the presence of functional β_2_-AR.

Sustained adrenergic overstimulation by elevated circulating catecholamines down regulates β-ARs, reducing the receptor density on the cell membrane and thus impairing downstream signaling. Seven day ISO treatment reduces the density of β-ARs, mainly β_2_-ARs, in cardiac tissue [54]. However, independent of changes in the number of β-ARs, the subtype β_2_-AR may uncouple from Gsα and couple to Giα protein; these effects are secondary to phosphorylation of β_2_-ARs or changes in the expression of G-protein isoforms [18], [55]. Thus, we investigated if the vascular effects of ISO treatment were associated with a down regulation of β-ARs in aortic tissue. It was previously shown that both β_1_- and β_2_-ARs were expressed in mouse conduit arteries [17], [21]. In contrast, no evidence of the β_3_-AR subtype was observed in these vessels [17], [21]. As expected, we found no expression of the β_3_-AR subtype in membrane extracts of mouse aorta, and ISO treatment had no effect. This suggested that the β_3_-AR subtype did not mediate the vascular effects of β-AR overactivation. β_1_- and β_2_-ARs were significantly expressed in the plasma membrane extracts of mouse aorta, but ISO treatment did not change their density in the membrane extracts from both WT and knockout mice. Thus, the vascular effects of ISO were not related to changes in β-AR subtype density; instead, the ISO effects were likely mediated by β_2_-AR coupling to up-regulated Giα protein.

It is known that chronic stimulation of β-AR recruits the proteins β-arrestins, that together G protein-coupled receptor kinases (GRKs) lead to desensitization of β-adrenoceptor, facilitating receptor internalization and can result in its degradation or recycling. In addition, β-arrestins and GRKs can play a role as signaling molecules mediating function other than the desensitization process. Considering that ISO stimulation of β_2_-AR can induce p-ERK in cultured HEK cells via β-arrestin and GRK 5/6 dependent and G protein-independent pathway [56], we cannot exclude the hypothesis that β-arrestin is involved in the isoproterenol-induced p-ERK 1/2 in vascular tissue, in addition to Gi protein pathway. On the other hand, haemodynamic alterations seems to be not involved in the vascular effect of chronic ISO administration, as previous report have demonstrated heart rate and arterial pressure in WT and β_2_KO treated for several days with ISO similar to WT group treated with saline [11], [57]. Of note, basal heart rate and arterial pressure in β_2_KO without ISO are not different from control WT [11], [21].

There is evidence for the potential clinical benefits of β_2_-AR signaling inhibition in cardiovascular diseases [58]–[60]. Importance of this signaling related to this beneficial effect was not clear because there is no available specific pharmacological tool to blockade Gi-coupled β_2_-AR signaling pathway [61]. Then, the genetic animal models are very helpful tools to reveal the functional importance of this signaling.

In conclusion, we propose a pivotal role of the β_2_-AR/Giα pathway in mediating the adverse effects of ISO-induced β-AR overstimulation. Enhanced p-ERK 1/2 and eNOS uncoupling lead to high superoxide anion production and reduced NO bioavailability, increasing the contractile response to phenylephrine in the mouse aorta. Prevalent diseases as heart failure and essential hypertension are particularly prone to vascular injury due to oxidative stress. In view of the central importance of sympathetic hyperactivity in these cardiovascular diseases, the involvement of β_2_-AR/Giα signaling pathway in adverse vascular effects provides new insight into therapeutic approach for improvement of endothelial dysfunction and NO bioavailability.
